# Safety, Acceptability, and Feasibility of Early Infant Male Circumcision Conducted by Nurse-Midwives Using the AccuCirc Device: Results of a Field Study in Zimbabwe

**DOI:** 10.9745/GHSP-D-15-00199

**Published:** 2016-07-02

**Authors:** Webster Mavhu, Natasha Larke, Karin Hatzold, Getrude Ncube, Helen A Weiss, Collin Mangenah, Prosper Chonzi, Owen Mugurungi, Juliet Mufuka, Christopher A Samkange, Gerald Gwinji, Frances M Cowan, Ismail Ticklay

**Affiliations:** a Centre for Sexual Health and HIV/AIDS Research (CeSHHAR), Harare, Zimbabwe; b University College London, London, United Kingdom; c London School of Hygiene & Tropical Medicine, London, United Kingdom; d Population Services International, Harare, Zimbabwe; e Ministry of Health and Child Care, Harare, Zimbabwe; f Harare City Health, Harare, Zimbabwe; g University of Zimbabwe College of Health Sciences, Harare, Zimbabwe

## Abstract

**Background:**

For prevention of HIV, early infant male circumcision (EIMC) needs to be scaled up in countries with high HIV prevalence. Routine EIMC will maintain the HIV prevention gains anticipated from current adult male circumcision initiatives. We present here the results of a field study of EIMC conducted in Zimbabwe.

**Methods:**

The study was observational and based on the World Health Organization (WHO) framework for clinical evaluation of male circumcision devices. We recruited parents of newborn male infants between August 2013 and July 2014 from 2 clinics. Nurse-midwives used the AccuCirc device to circumcise eligible infants. We followed participants for 14 days after EIMC. Outcome measures were EIMC safety, acceptability, and feasibility.

**Results:**

We enrolled 500 male infants in the field study (uptake 11%). The infants were circumcised between 6 and 60 days postpartum. The procedure took a median of 17 minutes (interquartile range of 5 to 18 minutes). Mothers’ knowledge of male circumcision was extensive. Of the 498 mothers who completed the study questionnaire, 91% knew that male circumcision decreases the risk of HIV acquisition, and 83% correctly stated that this prevention is partial. Asked about their community’s perception of EIMC, 40% felt that EIMC will likely be viewed positively in their community; 13% said negatively; and 47% said the perception could be both ways. We observed 7 moderate or severe adverse events (1.4%; 95% confidence interval, 0.4% to 2.4%). All resolved without lasting effects. Nearly all mothers (99%) reported great satisfaction with the outcome, would recommend EIMC to other parents, and would circumcise their next sons.

**Conclusion:**

This first field study in sub-Saharan Africa of the AccuCirc device for EIMC demonstrated that EIMC conducted by nurse-midwives with this device is safe, feasible, and acceptable to parents.

## Abstract (precis)

Early infant male circumcision (EIMC) conducted by nurse-midwives using the AccuCirc device proved safe, feasible, and acceptable to parents in Zimbabwe. The AccuCirc device has the potential to facilitate widespread scale-up of safe EIMC in sub-Saharan Africa.

## INTRODUCTION

Early infant male circumcision (EIMC) is simpler and less costly than voluntary medical male circumcision (VMMC).^1^^-^^3^ Also, EIMC is likely to prevent HIV acquisition more effectively than VMMC; EIMC is carried out long before an individual becomes sexually active and thus avoids the risk associated with sex during the healing period.^4^ In order for EIMC to maintain the HIV prevention gains anticipated through VMMC, it should be suitable for safe and efficient implementation in low-resource settings. Since large-scale EIMC has never been practiced in Southern Africa, demonstration of its safety, acceptability, and feasibility in this setting is crucial.

A number of circumcision devices are used with infants, including the Mogen clamp, Gomco clamp, and Plastibell.^5^ Each of these devices is associated with rare but potentially serious complications. Circumcision using the Mogen clamp can result in partial or total amputation of the glans penis or removal of too little foreskin (in which case the remaining foreskin remains vulnerable to infection with HIV).^5^^-^^8^ A mismatch in sizes of the separate pieces of the Gomco clamp can result in laceration of the glans penis.^5^^,^^9^ Proximal migration of the Plastibell during circumcision can result in necrosis of the glans and other injuries; this risk is increased if the incorrect size of “bell” is used.^5^^,^^10^^-^^12^

AccuCirc, a relatively new EIMC device (introduced in 2008), comes preassembled and thus may have the potential to address some of these shortcomings.^3^^,^^13^ In addition, it has a shielding ring that protects the glans penis, preventing laceration or amputation.^3^^,^^9^^,^^13^ The AccuCirc device is also disposable and does not require sterilization. These factors make it appealing for use in sub-Saharan Africa, particularly where health centers lack electricity and sterilization equipment.^3^^,^^9^ However, before AccuCirc is used to roll out EIMC in sub-Saharan Africa, further evidence is needed on its performance in comparison with other methods of EIMC.

Before AccuCirc is used to roll out EIMC in sub-Saharan Africa, further evidence is needed on its performance.

Although the World Health Organization (WHO) has determined that it will not prequalify medical devices for EIMC, the WHO “Framework for Clinical Evaluation of Devices for Male Circumcision”^14^ provides a useful and valid guide for evaluating novel EIMC devices for safety, acceptability, and feasibility. The guide recommends that, for innovative male circumcision methods, at least 3 successive studies be conducted in countries of intended use: a case series, a comparative trial, and a field study.^14^ Two studies to determine the safety, acceptability, and cost of the AccuCirc device in sub-Saharan Africa have been conducted in Botswana and Zimbabwe.^3^^,^^9^^,^^13^ In line with the WHO framework, Zimbabwe also conducted a field study of the AccuCirc device in which nurse-midwives performed EIMC. Here, we present the findings of this field study on safety, acceptability, and feasibility of the AccuCirc device for EIMC. Study findings will inform EIMC scale-up in Zimbabwe and the wider region.

## METHODS

### Study Design

The field study was observational. As the WHO “Framework for Clinical Evaluation of Devices for Male Circumcision”^14^ recommends, we enrolled, circumcised, and followed 500 participants. Participants were circumcised using the AccuCirc device and followed for 2 weeks post-circumcision.

### Recruitment and Training of EIMC Providers

In August and September 2013, doctors trained 4 study nurse-midwives over the course of 5 days to use the AccuCirc device. The trainers had previously performed circumcisions for the Zimbabwe EIMC comparative trial, described in detail elsewhere.^3^^,^^13^ The training curriculum consisted of didactic lectures, practical skill sessions, use of an EIMC anatomic model, written assessment, and practice evaluation. Trainees were required to score 100% on the written assessment and show competency using the anatomic model. Each provider then had to demonstrate competency performing 10 supervised circumcisions with the AccuCirc device.

### Participants

Mothers and infants were enrolled between August 2013 and July 2014 at Edith Opperman and Mabvuku, 2 polyclinics in Harare, with 400 and 250 deliveries per month, respectively.^15^ Women attending the clinic were informed of EIMC and enrolled at the antenatal clinic and after delivery in the maternity ward. In the community, we used educational materials (posters and pamphlets) and demand-creation activities (sensitization shows, dramas, and group and person-to-person discussions) to educate people about the field study. Before discharge postdelivery, mothers who were interested in having their male infants circumcised were asked to (1) provide locator information and consent for an outreach worker to physically verify their address, (2) complete an interviewer-administered questionnaire (asking, among other questions, about sociodemographic information and knowledge about HIV and male circumcision), and (3) discuss the procedure with the infant's father (if available) before attending for EIMC at their first postnatal visit.

We collected data on eligibility criteria from all mothers and infants. Maternal eligibility criteria were: (1) ability to attend follow-up appointments at the study clinic through 2 weeks postpartum, (2) readiness to provide a home address, and (3) written informed consent. Only infants ages 6 to 60 days were eligible. Although WHO guidelines^5^ state that infants can be circumcised as early as 12 to 24 hours old, we waited for 6 days to be sure that all body systems were stable and any immediate postpartum infant mortality would not be erroneously ascribed to EIMC. Additional infant eligibility criteria were: (1) male, (2) gestational age ≥ 37 weeks, (3) birth weight ≥ 2,500 grams, (4) no evidence of neonatal infection/sepsis or other illness requiring hospitalization, (5) no family history of bleeding disorder, and (6) no genital abnormality constituting a contraindication to EIMC. Between recruitment and enrollment, an outreach worker visited homes to verify the addresses of all potential field study participants.

### Intervention

Before enrollment, study staff performed a physical examination to exclude infants with abnormalities precluding circumcision. We referred to a specialist all 10 infants who were ineligible due to genital abnormalities. The 4 trained nurse-midwives (2 at each study clinic) performed all the circumcisions, assisted by 2 registered general nurses. To minimize bleeding, all infants received vitamin K (1 mg) an hour before the procedure. Additionally, all infants had approximately 1 gram of EMLA Cream (eutectic mixture of local anesthetics containing 2.5% lidocaine and 2.5% prilocaine) applied to the outer foreskin and shaft of the penis about 45 minutes before the procedure. Administration of vitamin K and the anesthetic is part of standard operating procedures for conducting EIMCs and not specific to the AccuCirc device.

We assessed achievement of anesthetic effect by holding the foreskin using artery forceps. If there was no pain response from the infant, subsequent steps would commence. Otherwise, providers would wait until the EMLA Cream had achieved anesthetic effect (an additional 10 to 15 minutes). When the EMLA Cream had achieved anesthetic effect, the surgical area was cleaned with povidone-iodine. Physiologic adhesions between the foreskin and the glans were released by manual technique using the flexible foreskin probe that comes with the AccuCirc kit. The nurse-midwife then marked the circumcision site (around the corona) with a surgical pen that is also part of each AccuCirc kit. The pen mark helps to minimize excessive or insufficient skin removal.

In addition to the flexible foreskin probe, the AccuCirc device consists of a shielding ring plus a single-action clamp (i.e., can be used only once) that contains a circular blade. It is available in 2 sizes: 1.1 cm and 1.3 cm (penile diameter).^9^ The nurse-midwives performed EIMC using AccuCirc following manufacturer’s recommendations (www.accucirc.com) and as recommended by the study previously conducted in Botswana.^9^ During the procedure, glucose water was given to the infant using a gloved finger, as has been recommended for pain management for neonatal circumcision.^15^^,^^17^^,^^18^ After the procedure, the circumcision wound was dressed, and infants were checked for post-procedure bleeding or other immediate complications. Nurse-midwives gave mothers detailed post-procedure care instructions (how to deal with dressing, bleeding, and signs of infection) and emergency contact information. Nurse-midwives also encouraged mothers to phone the nurse-coordinator or to come to the clinic if they had any worries or if unanticipated events occurred between scheduled visits.

### Follow-up and Evaluation

Follow-up appointments at the clinic took place at days 2, 7, and 14 after circumcision. At these visits, nurse-midwives asked parents about complications. The EIMC providers also conducted physical examinations of the infants, including inspection of the circumcision site. Seven days after the procedure we asked parents their views on the acceptability of circumcision.

### Outcome Measures

Outcome measures for the field study were (1) EIMC safety, (2) acceptability, and (3) feasibility. Safety was indicated by the number of moderate and severe adverse events (AEs). EIMC-related AEs were categorized as bleeding, infection, inadequate or excessive skin removal, or penile injury (glans, urethra, or shaft) (Table 1). We recorded minor events such as bleeding that could be stopped with simple compression, but excluded them from analyses. We measured EIMC acceptability by the proportion of parents who (1) adopted EIMC for their sons, (2) reported being satisfied with the procedure, and (3) expressed willingness to adopt EIMC for a future son.

**TABLE 1:** Classification of Procedure-Related Moderate and Severe Adverse Events

	Moderate	Severe
**Bleeding**	Bleeding that is not controlled by new dressings or 5 to 10 minutes of manual pressure and requires a special return to the clinic for a pressure dressing, additional skin sutures, or additional vitamin K administration without surgical re-exploration of the wound.	Bleeding that requires surgical re-exploration, hospitalization, or transfer to another facility, or any case where blood transfusion or intravenous fluid is necessary.
**Infection**	Discharge from the wound, painful swelling with erythema or elevated temperature,or use of oral antibiotics.	Cellulitis or abscess of the wound, or infection severe enough to require surgical intervention, hospitalization, or intravenous or intramuscular antibiotic therapy.
**Inadequate skin removal**	Prepuce partially covers glans when flaccid but surgical correction is not necessary.	Prepuce partially covers glans when flaccid and immediate surgical correction is necessary.
**Excessive skin removal**	Tightness of the skin discernible, and additional sutures or skin mobilizationneeded for wound closure, but no other intervention needed.	Reoperation or referral/transfer toanother facility required.
**Penile injury**	Significant laceration requiring either prolonged follow-up, care, and attention or repeated or additional dressings.	Significant injury including laceration or severed portion of glans, damage tothe urethra, or laceration of the shaft with ongoing bleeding that requires hospitalization, transfer, or transfusion.

We asked parents to rate their satisfaction with and acceptability of the procedure on a numerical scale ranging from 0 (dissatisfaction) to 10 (very satisfied). Satisfaction was defined as a score between 6 and 10. We also asked parents to rate on a scale of 0 to 10 the likelihood that they would recommend EIMC to friends or relatives. A score of 0 was classified as “would not recommend” and 10 as “would definitely recommend.” Feasibility was defined as spending the least amount of effort on a procedure and gauged by the time that the nurse-midwives needed to perform EIMC using AccuCirc (timed using a stopwatch plus video recording of each procedure), and the ability of nurse-midwives to safely perform the procedure. We considered15 to 30 minutes a “feasible procedure time,” based on the typical time spent in previous studies of the AccuCirc device.^9^^,^^13^

### Sample Size

We chose a sample size of 500 subjects, as specified by the WHO “Framework for Clinical Evaluation of Devices for Male Circumcision.”^14^ The rate of circumcision uptake was defined as the number of eligible male infants who were circumcised with AccuCirc, among those whose parents were asked to participate. With 500 infants undergoing EIMC, we could estimate a rate of 3% severe AEs with 95% confidence intervals (CIs) of 1.7% to 4.9%, and could therefore exclude a severe AE rate of greater than 5%.

### Statistical Analysis

We used Stata 13 (StataCorp, TX, USA) to perform statistical analyses. We summarized characteristics of parents and infants, including socio‐demographic characteristics, father’s reported circumcision status, and circumcision and HIV knowledge and attitudes. To evaluate the safety of the procedure, we calculated the proportion of procedures associated with AEs and the associated 95% CI. We calculated similar proportions and 95% CIs for the parental acceptability outcomes. To assess feasibility, we calculated the mean time for the procedure and an associated 95% CI.

### Ethical Considerations

The Medical Research Council of Zimbabwe and the ethics committees of University College London and the London School of Hygiene and Tropical Medicine approved the study. We obtained written informed consent from the infant’s mother and verbal consent from the infant’s father (if available) before enrollment. After the procedure, mothers received a 20-liter plastic bucket, 2 bars of washing soap, a 100-ml bottle of Vaseline (for wound care), and 3 disposable diapers (total value US$8). At each scheduled clinic visit, mothers also received the equivalent of US$5 for bus fare.

## RESULTS

### Participant Flow

To enroll 500 infants in the field study, we approached 4,617 parents of newborn male infants. Thus, the uptake rate was 11% (95% CI, 10.1% to 11.9%). Some 4,107 parents (89%) declined to participate. Reasons for refusal included fear of harm and sociocultural considerations.^19^ A further 10 male infants were excluded as ineligible (Figure). Between August 2013 and July 2014, 500 male infants ages 6 to 60 days were circumcised. The mother was in attendance during the procedure in 99.6% of cases. In the 2 instances when the mother was not there, the male infant’s father was present. Overall, fathers attended 34% of the male infant circumcisions (Table 2). All but 1 participant (described in detail later) attended all 3 scheduled follow-up visits on days 2, 7, and 14.

**FIGURE:**
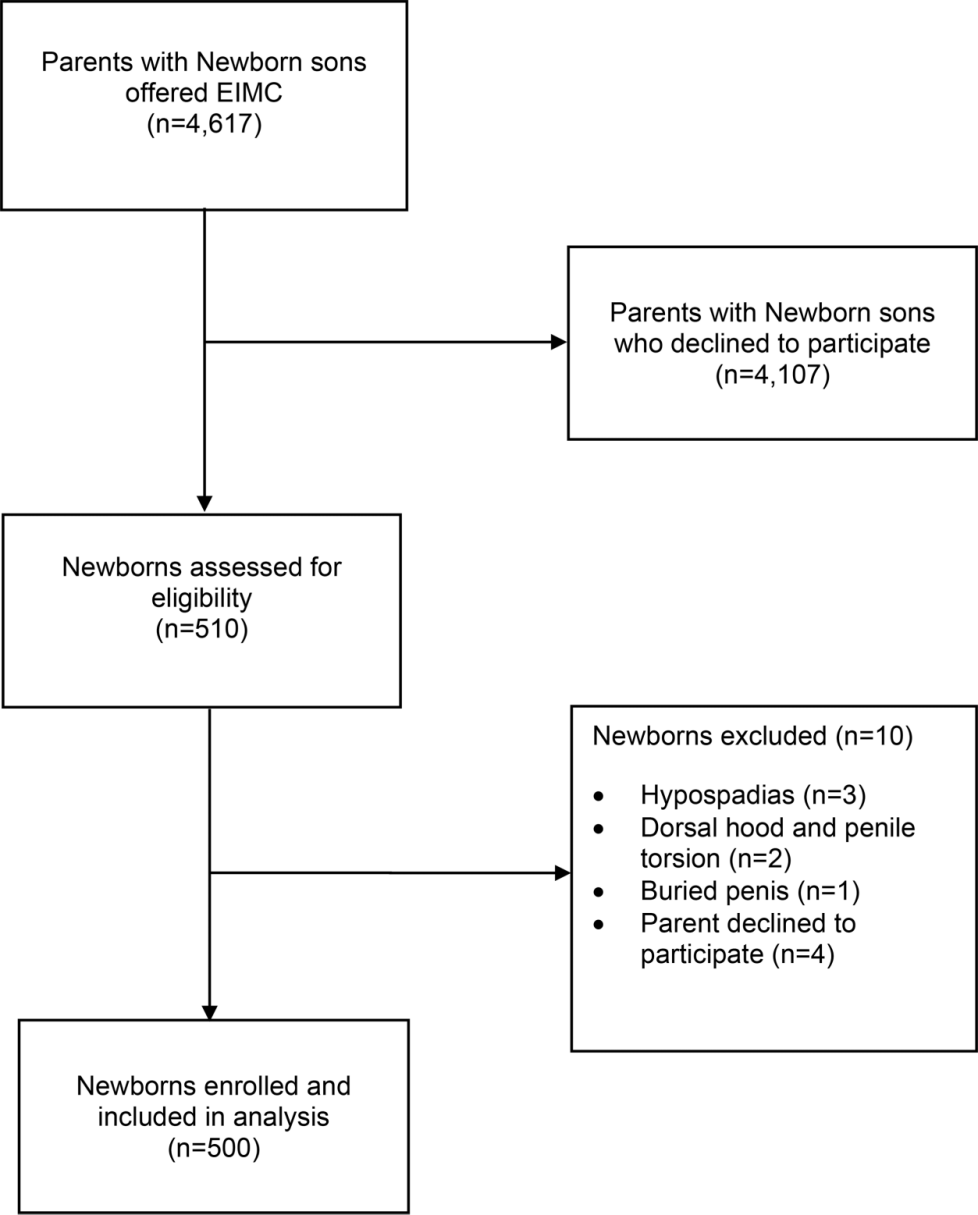
Recruitment of Participants in the EIMC Field Study Abbreviation: EIMC, early infant male circumcision.

**TABLE 2:** Characteristics of Infants and Mothers (N = 500 Infants and 500 Mothers)

Characteristics and Views	
**Infants**	
Age, days, median (IQR)	22 (12, 46)
Birth weight, kg, median (IQR)	3.2 (2.9, 3.5)
Temperature, Celsius, median (IQR)	36.2 (36.0, 36.5)
Gestational age, weeks, median (IQR)	40 (38, 40)
Vitamin K given, No. (%)	500 (100)
HIV exposure status,^a^ No. (%)	85 (17)
Mother present,^b^ No. (%)	498 (99.6)
Father present, No. (%)	168 (34)
**Mothers**	
Mother’s age, years, No. (%)	
≤20	39 (8)
21–25	123 (25)
26–30	159 (32)
31–35	123 (25)
>35	56 (11)
Current marital status, No. (%)	
Married	478 (96)
Not married	21 (4)
Widowed	1 (0.2)
With whom do you live?,^c^ No. (%)	
Alone	9 (43)
Male partner	1 (5)
Other	11(52)
Completed secondary level education?, No. (%)	
No	205 (41)
Yes	295 (59)
Ethnic group, No. (%)	
Shona	406 (81)
Non-Shona	94 (19)
Head of household,^d^ No. (%)	
Mother	28 (6)
Male partner	431 (91)
Someone else	14 (3)
Infant's father circumcised?, No. (%)	
Yes	187 (39)
No	273 (58)
Don’t know	13 (3)
HIV knowledge score,^e^ median (IQR)	6 (4, 6)
Circumcision knowledge score,^f^ median (IQR)	6 (5, 8)
Heard of male circumcision as an HIV prevention method before today, No. (%)	431 (91)
Does male circumcision really protect men against HIV?, No. (%)	
Completely	23 (5)
Partially	393 (83)
No, it does not protect	15 (3)
Don’t know	42 (9)
Would like male partner to be circumcised,^g^ No. (%)	219 (80)
Who should perform EIMC?, No. (%)	
Trained doctors	433 (92)
Trained nurse-midwives	24 (5)
Traditional leader of the same tribe or religion	4 (1)
Other	9 (2)
How will EIMC likely be viewed in community?, No. (%)	
Negatively	63 (13)
Positively	185 (40)
Both negatively and positively	221 (47)

Abbreviations: EIMC, early infant male circumcision; IQR, interquartile range.

a HIV status unknown for 4 infants.

b In the 2 cases where the mother was not present, the infant’s father was present during the procedure.

c Among the 22 not married or widowed mothers, 1 mother did not give an answer.

d Data collected from only 473 mothers.

e HIV knowledge score composed of 8 questions, with 1 point for every correct answer (maximum score  =  8).

f Circumcision knowledge score composed of 8 questions, with 1 point for every correct answer (maximum score  =  8).

g Among 273 mothers who reported their partner was not currently circumcised.

### Characteristics of Enrolled Infants and Their Mothers

The median age of male infants at the time of circumcision was 22 days (interquartile range [IQR], 12 to 46) (Table 2). Eighty-five (17%) of the infants were exposed to HIV. The median age of mothers was 28 years (IQR, 24 to 32). The majority of mothers were married (n = 478, 96%), and more than half had completed secondary education (n = 295, 59%). Approximately 40% of fathers were reportedly circumcised. Among mothers whose partner was not circumcised, 80% stated they would like him to be circumcised. Knowledge of circumcision was high among mothers who completed the study’s quantitative questionnaire, with 431 (91%) mothers reporting that they had heard of the preventive benefits of circumcision for HIV, and 393 (83%) correctly stating that circumcision was partially protective against HIV. Some 185 mothers (40%) felt that EIMC will likely be viewed positively in their community; 63 (13%) said negatively; and 221 (47%) said both ways. Even though they were largely satisfied by the procedure performed by nurse-midwives, a majority of mothers (n = 433, 92%) thought trained doctors should perform EIMC.

Approximately 40% of fathers were reportedly circumcised. Among mothers whose partner was not circumcised, 80% would like their partner to be circumcised.

### Procedure Time and Intraoperative Events

Completing the procedure took a median of 17 minutes (IQR, 15 to 18). Mild post-circumcision bleeding occurred in 49 male infants (10%). For 46 of these infants (94%), simple pressure stopped this bleeding. Three cases of bleeding could not be stopped by simple pressure. We classified these as moderate or severe procedure-related AEs; we detail these cases below.

Completing the procedure took a median of 17 minutes.

### Adverse Events

We observed 7 AEs (1.4%; 95% CI, 0.4% to 2.4%) (Table 3). These included 2 cases of excessive removal of skin (moderate severity, requiring skin mobilization; wound completely healed 4 months post-EIMC in both cases) and 2 cases of inadequate skin removal (moderate severity; urologist concluded that neither required immediate corrective surgery), 1 case of moderate bleeding, and 2 cases of severe bleeding. There were no sequelae in any of the mild, moderate, or severe AE cases.

Moderate or severe adverse events occurred in 7 of 500 circumcisions.

**TABLE 3:** Adverse Events Associated With EIMC (N = 500), Time Taken for the Procedure, and Parental Satisfaction

Outcome	
All AEs, No. (%)	7 (1.4)
Moderate/severe bleeding	3 (0.6)
Infection	0 (0.0)
Inadequate skin removal	2 (0.4)
Excess skin removal	2 (0.4)
Injury to penis	0 (0.0)
Time taken to perform procedure, minutes, median (IQR)	17 (15, 18)
**Mothers’ satisfaction (N = 498**^a^ **)**	
Satisfaction score (0–10), No. (%)	
0–5	0 (0)
6–7	7 (1)
8	23 (5)
9	26 (5)
10	442 (89)
Reasons for dissatisfaction, No. (%)	
Appearance	22 (4)
Wound care requirements	23 (5)
Complication	2 (0.4)
Other reason	13 (3)
Mother would definitely recommend MC,^b^ No. (%)	496 (99)
Mother would have a future son circumcised, No. (%)	497 (99)
**Fathers’ satisfaction (N = 112)**	
Satisfaction score (0–10), No. (%)	109 (97)
0–4	0 (0)
5–6	5 (4)
7–9	15 (13)
10	92 (82)
Reason for dissatisfaction, No. (%)	
Appearance	3 (3)
Wound care requirements	9 (8)
Complication	1 (1)
Other	7 (6)
Father would definitely recommend MC,^b^ No. (%)	109 (98)
Father would have a future son circumcised, No. (%)	110 (98)

Abbreviations: AE, adverse event; EIMC, early infant male circumcision; IQR, interquartile range; MC, male circumcision.

a Two mothers did not provide information on their satisfaction with the procedure.

b Parental recommendation score of 10 (out of 10).

The 1 case of moderate bleeding required clamping of the bleeding vessel with forceps and an acetylated glucosamine dressing at the time of the procedure. The 2 cases of severe bleeding required hospitalization. In 1 case, the infant bled immediately post-circumcision. The bleeding was controlled with pressure but started again at home, and he was referred to a hospital. Three sutures were required to stem the bleeding. The other case of severe bleeding was in an infant who had a family history of hemophilia (unreported and undetected at the time of the procedure). The infant was given a blood transfusion (200 ml). He was hospitalized for 3 days.

### Unrelated Newborn Death

One male infant left the study 2 days after the procedure without any formal follow-up visits. This infant developed an acute respiratory infection after the procedure was performed and died 36 hours post-EIMC. The respiratory infection was due to an outbreak of severe bronchiolitis and was deemed unrelated to the EIMC procedure.

### Parental Satisfaction

All mothers who answered the question on satisfaction with EIMC (n = 498) reported being satisfied with the outcome (scores  =  6 to 10), with 491 (99%) reporting that they were very satisfied (scores  =  8 or greater). Nearly all mothers (n = 496, 99%) said they would recommend EIMC to other parents and would circumcise their next newborn son (n = 497, 99%). Among fathers who answered the question on satisfaction with EIMC, all 112 gave a score of 6 or greater. Nearly all the fathers would recommend the procedure to a friend (n = 109, 98%) and would have a future son circumcised (n = 110, 98%) (Table 3). The most common reason for dissatisfaction, cited by 5% of mothers and 8% of fathers, was the wound care requirements.

## DISCUSSION

We have implemented the first field study in sub-Saharan Africa of EIMC conducted by nurse-midwives using the AccuCirc device. We found that the procedure is safe, feasible, and acceptable to parents who choose it for their infant sons.

Overall, the rate of AEs was low and similar to those reported in the 2 previous AccuCirc studies conducted in Botswana^9^ and Zimbabwe.^13^ In both of these studies, doctors performed the circumcisions. The Botswana study identified 1 moderate AE (0.7%; 95% CI, 0.1% to 4.6%), and the Zimbabwe study identified 2 moderate AEs (2%; 95% CI, 0.2% to 7.0%).^9^^,^^13^ Thus, our findings confirm previous findings that EIMC is a simple and safe procedure, characterized by a low rate of AEs.^2^^,^^6^^,^^9^^,^^20^^,^^21^ Moreover, AE rates were similar between the nurse-midwives in this study (1.4%; 95% CI, 0.4% to 2.4%) and the doctors in the Zimbabwe study (2%; 95% CI, 0.2% to 7.0%).^13^ This similarity suggests that it is both safe and feasible to roll out EIMC using non-doctor providers—a particularly important conclusion for sub-Saharan Africa, where nurses and midwives are in far greater supply than doctors.^3^^,^^9^^,^^22^ Despite accepting EIMC delivered by nurse-midwives, 92% of mothers thought that the procedure should be performed by trained doctors. Because this is a widespread preference, the need to educate parents on EIMC safety in the hands of nurse-midwives and other trained non-physician providers is a priority.^23^

Similar AE rates for physicians and nurse-midwives suggest that it is safe and feasible to roll out EIMC using non-physician providers.

Nonetheless, the fact that we encountered 2 cases of severe bleeding (including a case of unreported hemophilia) that required hospitalization for monitoring and management has important implications for EIMC planning and subsequent roll-out:

1. Although EIMC can be offered at the lowest-level health facilities, there should be easily accessible backup services to deal with any life-threatening complications (e.g., excessive bleeding).
2. Intensive screening and history-taking before the procedure are required in order to exclude any infants with a family history of bleeding disorder.
3. Screening for other non-circumcision-related health issues is necessary to ensure that infants who are circumcised are clinically well.

The infant mortality rate is high in sub-Saharan Africa. Recent Zimbabwe Demographic and Health Survey findings suggest that infant and neonatal mortality rates are 57 and 31 deaths per 1,000 live births, respectively.^24^ Although the EIMC procedure itself is unlikely to result in major morbidity or mortality, it is important that babies who are unwell with other conditions are not circumcised. Such events may be mistakenly ascribed to EIMC.^9^ It will therefore be crucial to offer EIMC when all body systems are stable so that any immediate postpartum infant mortality is not erroneously ascribed to EIMC.

As in the Zimbabwe comparative trial,^13^ actual uptake of the procedure, at 11%, was far lower than the 60% hypothetical acceptability that other studies had predicted.^23^^,^^25^ Previous studies in Zimbabwe and Zambia^13^^,^^26^ reported similar discrepancies. However, in both Zambia and Zimbabwe, EIMC was still offered in a research setting. The rate of EIMC uptake may differ when it is offered as part of an ongoing program.^13^ Zimbabwe is traditionally a non-circumcising country. It is to be expected that it will take time, and the program will need to earn the trust of parents, before EIMC—now an unfamiliar and perhaps scary procedure—becomes accepted.^13^ Nonetheless, culturally appropriate demand-creation activities to promote EIMC need to be conducted if EIMC is to become the norm.^13^

Actual uptake of the procedure—11%—was lower than previously suggested by hypothetical acceptability studies.

Sustained uptake and acceptability will depend greatly on perceptions of the safety and aesthetic aspects of EIMC.^27^ Encouragingly, in this field study nearly all mothers (99%) reported high satisfaction with the outcome. These findings are consistent with others from the region (Botswana, Kenya, and Zambia), which report levels of satisfaction over 90%.^6^^,^^10^^,^^28^ With specific reference to satisfaction with EIMC performed via AccuCirc, the findings are consistent with those from our comparative trial and from Botswana, where over 91% of mothers reported high or complete satisfaction with the outcome.^9^^,^^13^ To maintain these high levels of satisfaction, EIMC provision will need to be carefully monitored and supervised to ensure that AEs are minimized and appropriately managed.^23^ As with most self-reported data, the high levels of satisfaction could be due to social desirability bias. However, after qualitatively exploring this issue^29^ and triangulating findings from different data collection approaches, we can safely conclude that the reported levels of satisfaction are a true reflection of what parents actually felt.

Most women who sought EIMC for their sons were knowledgeable about male circumcision and its HIV-preventive benefits. Our findings corroborate those from previous studies on the hypothetical acceptability of VMMC and EIMC; these studies found that this knowledge is likely to be associated with uptake.^23^^,^^30^ Widespread awareness campaigns to enhance men’s and women’s knowledge of male circumcision and its benefits will be essential for successful scale-up. Furthermore, about 40% of women who sought EIMC for their sons reported that the infant's father was circumcised, whereas the prevalence of male circumcision in the general male population is estimated to be around 10%.^24^ This suggests that, as adult VMMC spreads, demand for EIMC is likely to grow.

A few women (n = 63, 13%) felt that EIMC will likely be viewed negatively in their community. Hypothetical acceptability studies have suggested that fear of their son’s future social exclusion, including ostracism, derision, and rejection, may discourage parents from opting for EIMC.^30^^-^^32^ Current initiatives that portray VMMC as a lifestyle choice for a man who is clean, elegant, and confident are likely playing a critical role in changing community norms about the procedure.^33^ In addition, uptake of VMMC in Zimbabwe continues to rise (from 10,000 in 2009 to 600,000 as of December 2015); community norms are also likely to change as more men opt for circumcision.

Current initiatives that portray VMMC as a lifestyle choice likely are playing a critical role in changing community norms.

EIMC may provide a rare opportunity to reach fathers via health care facilities. In this study, for example, about one-third of fathers accompanied their sons to the EIMC clinic. Otherwise, men are known to be hard to reach via health services.^34^^-^^36^ Additionally, men in general, and Zimbabwean men specifically, rarely accompany their wives to the clinic for antenatal or infant care. But it may be possible to use EIMC as a platform to strengthen other maternal, newborn, and child health programs and to promote other health interventions to men, such as VMMC and prevention of mother-to-child transmission of HIV.

It may be possible to use EIMC as a platform to strengthen other maternal and child health programs and to promote other health interventions to men.

### Limitations

The sample size of this field study was guided by the WHO “Framework for Clinical Evaluation of Devices for Male Circumcision,”^14^ but the number was nonetheless small. Thus, it is possible that we did not detect all potential AEs that might occur during EIMC roll-out. Field studies of the AccuCirc device currently under way in Kenya will increase our knowledge of AEs associated with the procedure. We also studied EIMC only in an urban setting. Findings, particularly on acceptability, might be different in rural areas. Similar research needs to take place in a different setting in order to further inform EIMC programming and roll-out.

## CONCLUSIONS

We circumcised 500 male infants in a field study of the AccuCirc device for EIMC in Zimbabwe. We found that EIMC can be safely and acceptably offered by nurse-midwives using the AccuCirc device. The AccuCirc device has the potential to facilitate widespread scale-up of safe EIMC in sub-Saharan Africa.

## Notes

**Peer Reviewed**
